# Rapid, ultraviolet-induced, reversibly switchable wettability of superhydrophobic/superhydrophilic surfaces

**DOI:** 10.3762/bjnano.10.87

**Published:** 2019-04-15

**Authors:** Yunlu Pan, Wenting Kong, Bharat Bhushan, Xuezeng Zhao

**Affiliations:** 1 Key laboratory of Micro-Systems and Micro-Structures Manufacturing of Ministry of Education and School of Mechatronics Engineering, Harbin Institute of Technology, Xidazhi 92, Harbin, 150001, PR China; 2 Nanoprobe Laboratory for Bio- & Nanotechnology and Biomimetics (NLBB), The Ohio State University, 201 W. 19th Avenue, Columbus, OH 43210-1142, USA

**Keywords:** superhydrophilic surfaces, superhydrophobic surfaces, switchable wettability, TiO_2_, trimethoxy(alkyl)silane, UV illumination

## Abstract

Controllable wettability is important for a wide range of applications, including intelligent switching, self-cleaning and oil/water separation. In this work, rapid switching and extreme wettability changes upon ultraviolet (UV) illumination were investigated. TiO_2_ nanoparticles were modified in solutions of trimethoxy(alkyl)silane, and the suspensions were sprayed on glass substrates. For such samples, the water contact angle (WCA) was shown to transition from a superhydrophobic (WCA ≈ 165°) to a superhydrophilic (WCA ≈ 0°) state within 10 min upon UV illumination and subsequent recovery to superhydrophobicity occurred after heat treatment. It was found that the changes in the trimethoxy(alkyl)silane upon UV illumination can explain the rapid decrease of the WCA from more than 165° to almost 0°. To further investigate the wettability transition, trimethoxy(alkyl)silane and Al_2_O_3_ nanoparticles (which are not photocatalytic) were mixed and spray-coated onto the glass substrates as the control samples. Then the unrecoverable change of trimethoxy(alkyl)silane under UV illumination can be confirmed. It was found that the presence of trimethoxy(alkyl)silane in the TiO_2_–trimethoxy(alkyl)silane coating served to speed up the super-wettability transition time from superhydrophobicity to superhydrophilicity, but also limited the number of wettability recycle times. With this understanding, the effect of the trimethoxy(alkyl)silane concentration on the number of recycle cycles was investigated.

## Introduction

Wettability is an important property of solid surfaces governed by surface chemistry and surface topography [1–2] and has found significant applications in various fields [3–8]. Controllable wettability that can be enabled through external stimuli, such as illumination, electric fields or heating, can be applied in chemical sensors, smart filtration and separation, and microfluidic devices [9–12]. While controlling wettability through heating is mostly limited to toxic materials, such surfaces cannot be applied in human science [13–14]. Although the application of an electric field is an efficient method to achieve switchable wettability, the range of variation in the wettability is still limited [15–16]. Among the numerous approaches to achieve switchable wettability, UV illumination is highly preferred due to its environmentally friendly, non-contact and convenient manner. TiO_2_ nanoparticles are a common photocatalysis material that has attracted attention since it is more sensitive to UV light [17]. The use of a photochemical method to strengthen the interaction between nanoparticles and organic materials is quite common [18–19]. TiO_2_ nanoparticles modified with organic materials that have a low surface energy could be used to induce the property of switchable wettability under UV illumination.

In order to be useful for many applications, the ability to rapidly switch the wettability from superhydrophobicity to superhydrophilicity is imperative. Several studies based on TiO_2_ have been carried out to prepare surfaces exhibiting reversible wettability [20]. Wang et al. [21] prepared a TiO_2_ polycrystalline film which achieved drastic changes in wettability when the water contact angle (WCA) was changed from 72° and 0° under UV illumination, followed by recovery at around 70° after storing in the dark. Jin et al. [22] modified TiO_2_ with 7-[4-(trifluoromethoxyphenylazo)phenoxy]pentanoic acid (CF_3_AZO) which could reversibly switch between hydrophobic (≈145°) and hydrophilic (≈24°) under UV illumination for about 13 h. Chagas et al. [23] fabricated surfaces that were reversibly wettable by dipping polypropylene surfaces in a suspension of TiO_2_ modified with trimethoxypropyl saline. In this work, the wettability transition time from superhydrophobic (WCA ≈ 158°) to superhydrophilic (WCA ≈ 0°) occurred within 120 min of UV illumination.

Although large range wettability switching can be achieved in many ways, the transition from superhydrophobic to superhydrophilic always requires a longer time, ranging from a couple of days to a couple of hours [23–27]. Some studies have been carried out to reduce the wettability transition time. Sawada et al. [28] fabricated surfaces with TiO_2_/fluoroalkyl end-capped vinyltrimethoxysilane sol–gel where the WCA changed from about 180° to 108° in under 50 min upon UV illumination and then further reduced to 20° after another 30 min. Thus the total transition time from superhydrophobic to superhydrophilic was about 80 min. Petroffe et al. [29] modified TiO_2_ with 11-(4-(phenylazo)phenoxy)undecanoic acid (AzoC11) acid to fabricate a hybrid surface which could achieve a rapid change in wettability properties within 12 min, however the wettability range was only between hydrophobic (≈146°) and hydrophilic (≈21°). Qing et al. [30] fabricated superhydrophobic TiO_2_ nanoparticles with (heptadecafluoro-1,1,2,2-tetradecyl)trimethoxysilane, which not only exhibited reversible wettability from 160° to 0° but also reduced the transition time to 60 min.

By modifying trimethoxy(alkyl)silane, TiO_2_-based surfaces can achieve a faster change from superhydrophobic to superhydrophilic. However, the reason of the faster transition process has not explained. Due to the high energy of UV illumination and the photocatalysis effect of TiO_2_, the –CF_2−_, –CF_3_ groups of the modified trimethoxy(alkyl)silane might become photodegraded and the end of the trimethoxy(alkyl)silane would change into –OH groups without reversibility [31–32]. It can then be assumed that the degradation of trimethoxy(alkyl)silane could reduce the transition time from superhydrophobic to superhydrophilic for surfaces modified with TiO_2_ nanoparticles and trimethoxy(alkyl)silane.

In this study, to confirm this assumption, a series of experiments were carried out. Surfaces were coated with TiO_2_ nanoparticles and non-photosensitive Al_2_O_3_ nanoparticles as the control groups, respectively. The nanoparticles were modified with a short carbon chain trimethoxy(alkyl)silane, 1*H*,1*H*,2*H*,2*H*-perfluorooctyl(trimethoxy)silane (PFOS), in different concentrations. The wettability and the chemical bonding of the surfaces were tested upon UV illumination and heating treatments. The results are in agreement with the proposed assumption that the oxidization of the trimethoxy(alkyl)silane could decrease the super-wettability transition time, however the reversibility will be limited due to the irreversible oxidization process. The effect of trimethoxy(alkyl)silane concentration on the number of times the surface can be recycled was also investigated.

## Results and Discussion

### Characterization of the superhydrophobic coating

**Figure 1:**
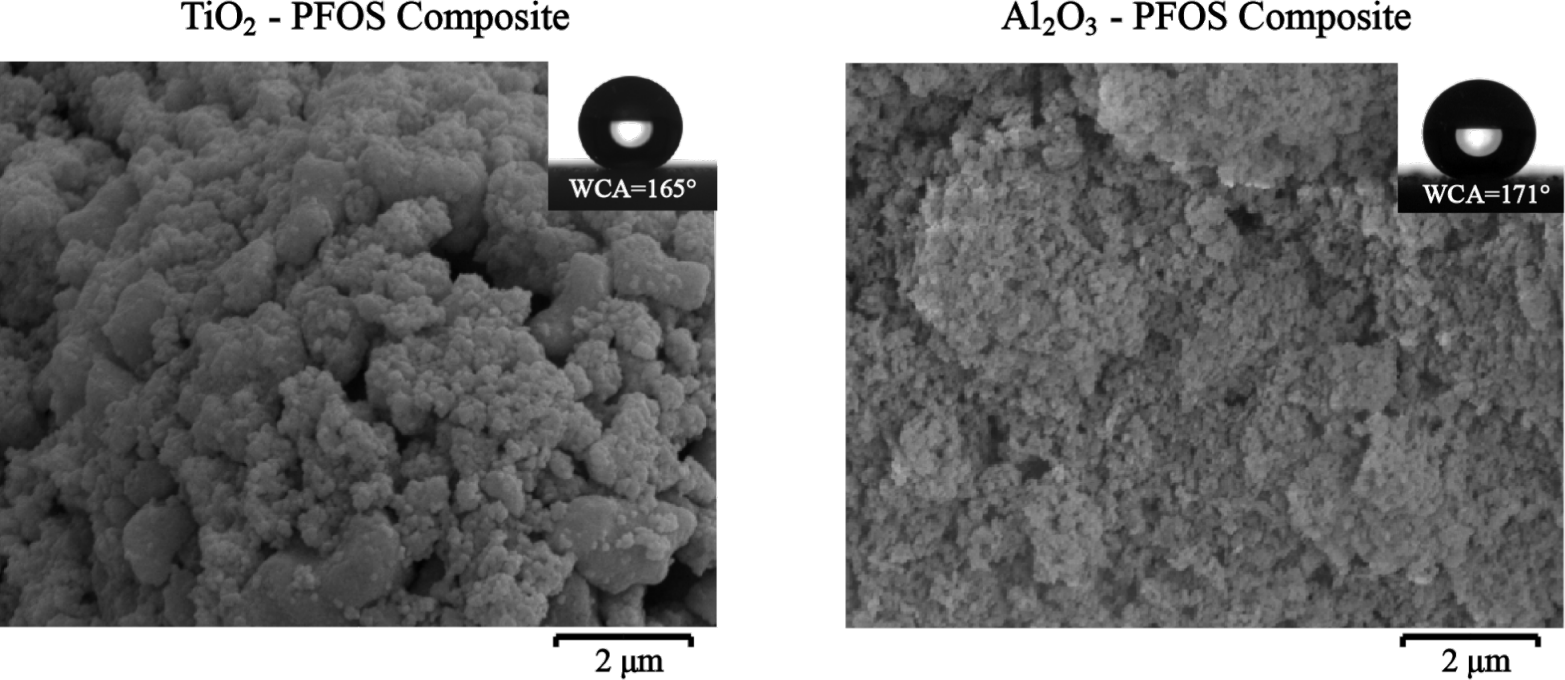
SEM images of TiO_2_–PFOS and Al_2_O_3_–PFOS composite surfaces.

The hydrophilic TiO_2_ nanoparticles were modified in ethanol solutions of PFOS, and the suspensions were sprayed on glass substrates to make the TiO_2_–PFOS samples. With the same method, Al_2_O_3_–PFOS samples were prepared as control groups. As shown in Figure 1, both TiO_2_–PFOS and Al_2_O_3_–PFOS coated surfaces exhibited excellent superhydrophobicity, and the WCA was measured as about 165° and 171°, respectively. Based on the SEM images shown in Figure 1, two-dimensional micro–nanostructures can be obviously seen on the two kinds of surfaces due to the aggregation of nanoparticles, which can explain the good superhydrophobicity.

**Figure 2:**
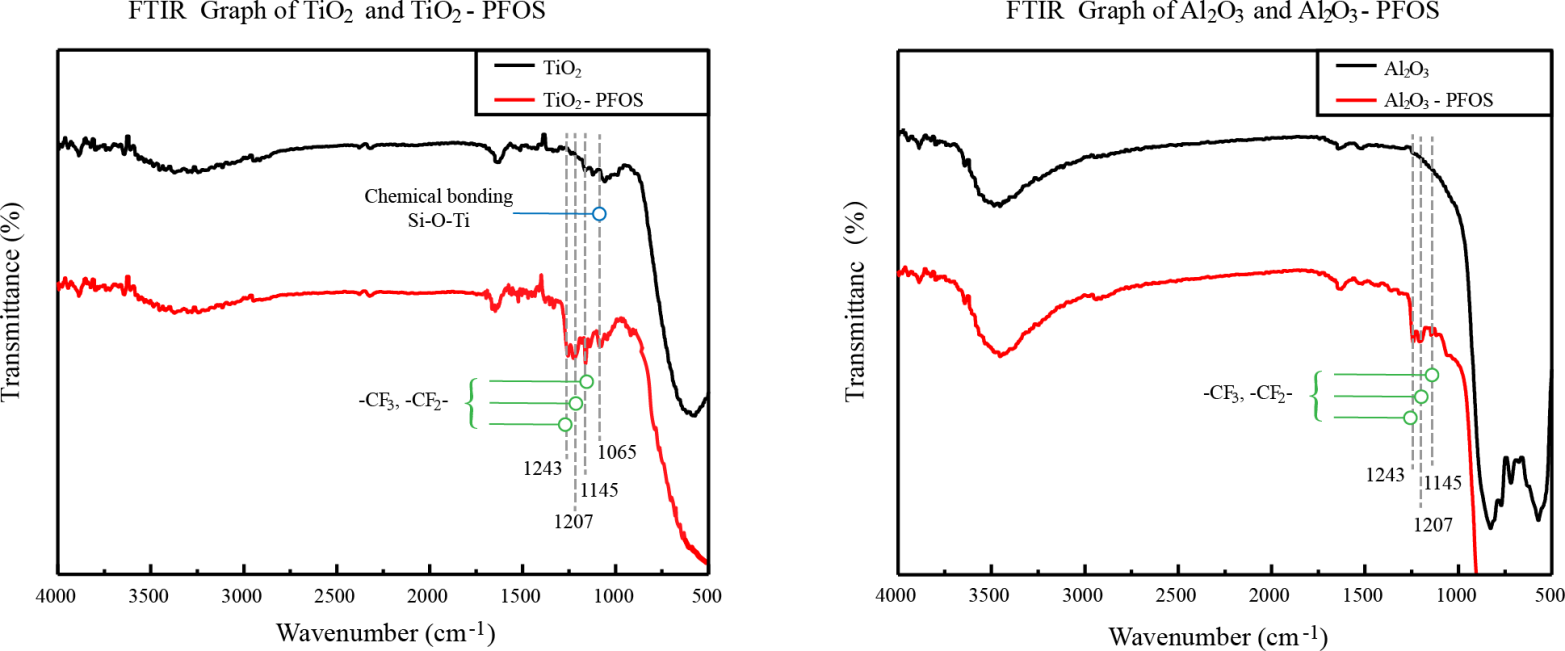
FTIR spectra of TiO_2_ and TiO_2_–PFOS, Al_2_O_3_ and Al_2_O_3_–PFOS.

The TiO_2_ nanoparticles were subjected to chemical bonding as Si–O–Ti with hydrolysing PFOS. The chemical bonding is verified by the Fourier transfer infrared (FTIR) spectra of TiO_2_ and TiO_2_–PFOS coated glass surfaces, as shown in Figure 2. The asymmetric stretching vibration of the Si–O–Ti species was displayed at the absorption peak of 1065 cm^−1^ which further confirmed the dehydration reaction occurred between the hydrolytic PFOS and TiO_2_. Additionally, there were another three peaks at 1157, 1207, 1243 cm^−1^, which correspond to the stretching vibration of –CF_2−_ and –CF_3_ groups [24,30]. Similarly, the absorption peak of –CF_2−_ and –CF_3_ groups appeared in the FTIR spectra of Al_2_O_3_–PFOS but without the peak at 1065 cm^−1^, which indicates that there was probably no chemical bonding between Al_2_O_3_ and PFOS, suggesting the physical adhesion between Al_2_O_3_ nanoparticles and PFOS.

### Wettability switching by UV illumination and heating

**Figure 3:**
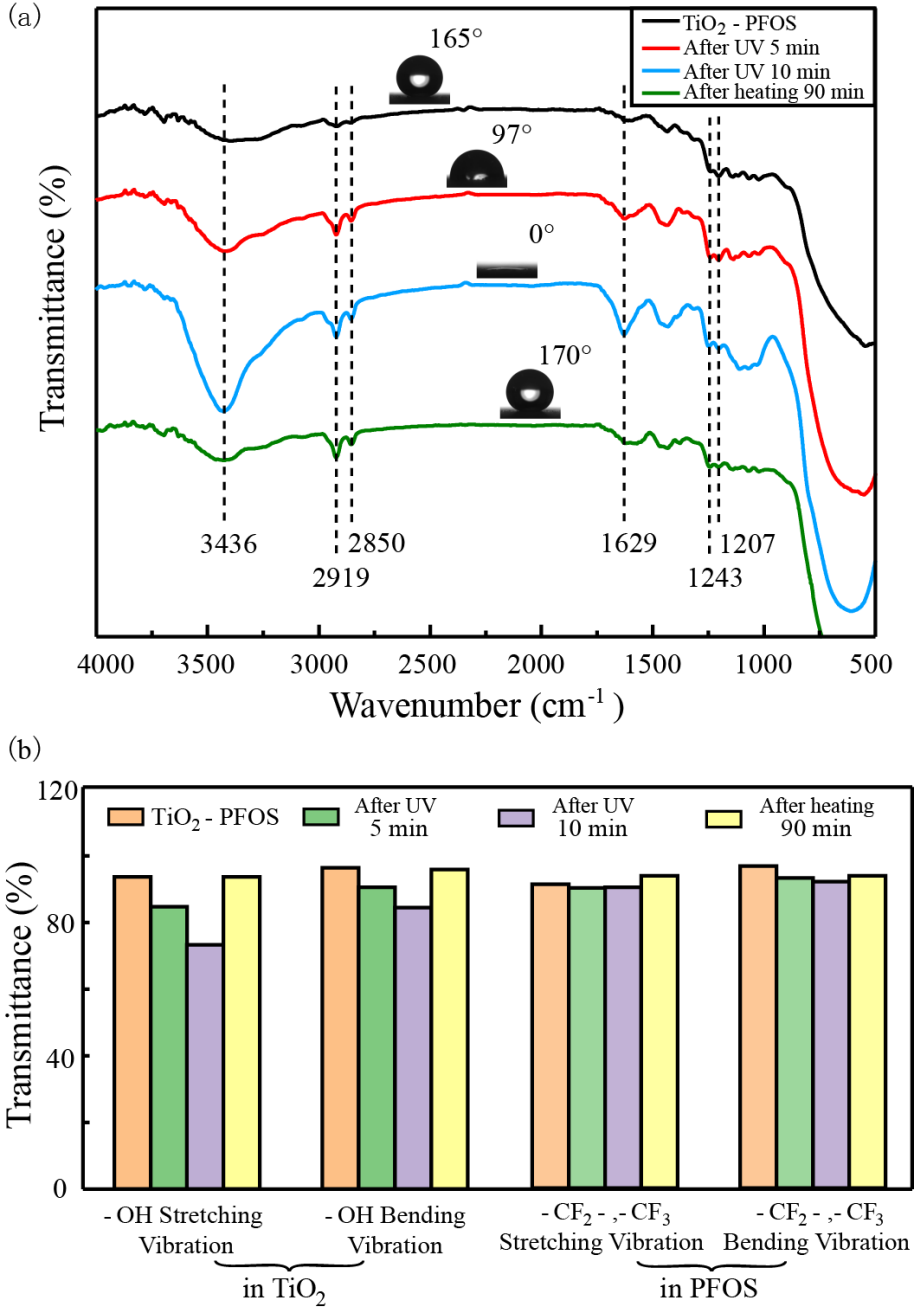
(a) Contact angle and FTIR spectrum of the TiO_2_–PFOS surface under different treatment conditions. (b) The change in transmittance for selected transitions, –OH, –CF_2_- and –CF_3_.

**Figure 4:**
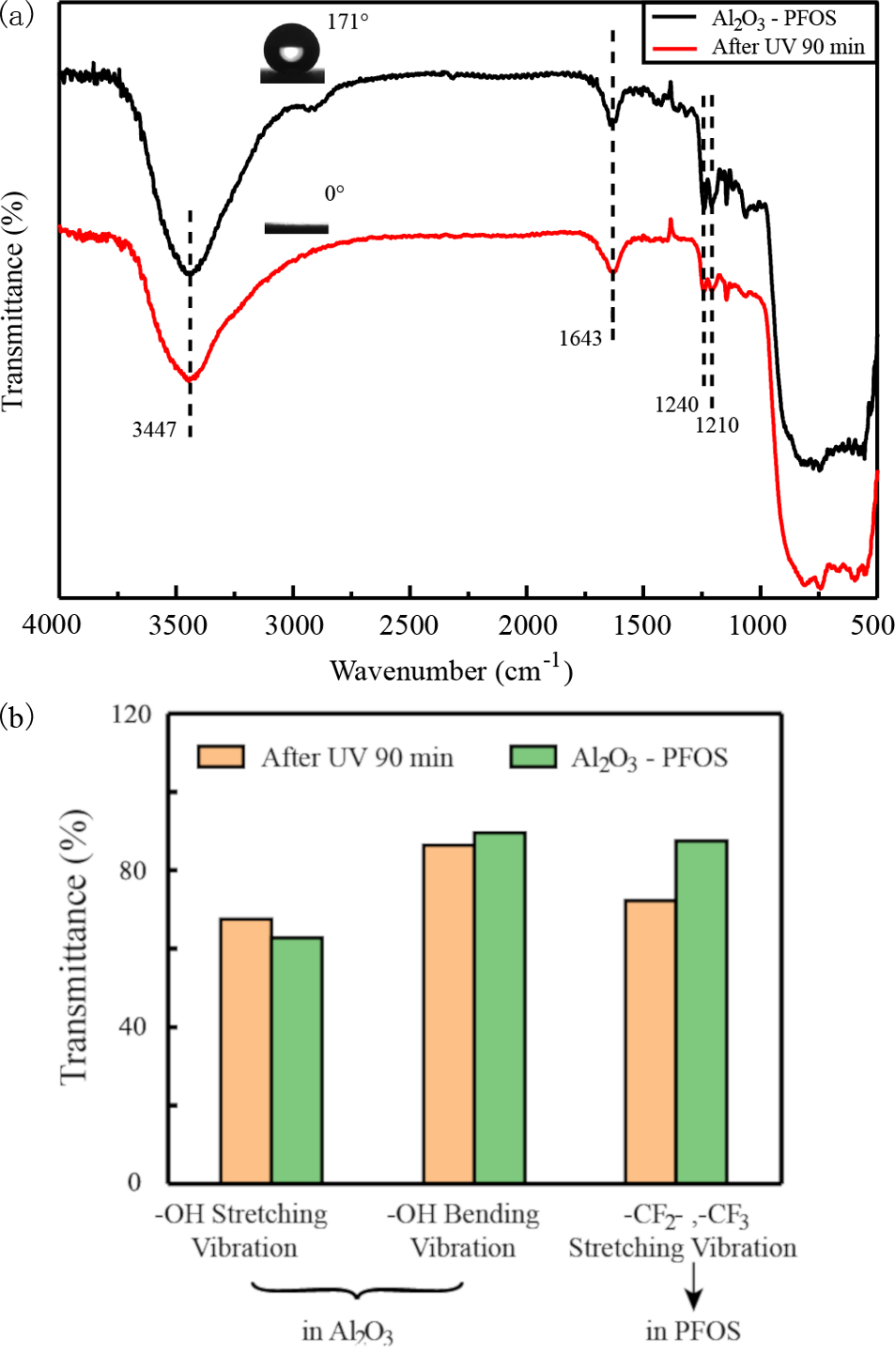
(a) Contact angle and FTIR spectrum of the Al_2_O_3_–PFOS surface under different treatment conditions. (b) The change in transmittance for selected transitions, –OH, –CF_2_- and –CF_3_.

Both TiO_2_–PFOS coated surfaces and Al_2_O_3_–PFOS coated surfaces were directly illuminated under a UV lamp at a working distance of 10 mm, and the WCAs were recorded every 5 min. The TiO_2_–PFOS coated surface transitioned from superhydrophobicity to superhydrophilicity after only 10 min of UV exposure, while it took 90 min for Al_2_O_3_–PFOS surfaces to transition. Then the samples were heated at 150 °C for 90 min, the property of superhydrophobicity was recovered for the TiO_2_–PFOS coated surface, while the Al_2_O_3_–PFOS coated surface remained superhydrophilic. A transition time of 10 min from superhydrophobic to superhydrophilic is much faster than in previous works [22–27]. The samples in the process of wettability switching were observed by FTIR, as shown in Figure 3a. For the TiO_2_–PFOS surface, as the UV exposure time increases, the peak intensity at 1629 cm^−1^ and 3436 cm^−1^ (assigned to the bending vibration and stretching vibration of –OH groups, respectively) increased, while both peaks reduced after the heating treatment. At the same time, four peaks at 1207 cm^−1^, 1243 cm^−1^, 2850 cm^−1^ and 2919 cm^−1^ (attributed to the bending vibration and stretching vibration of –CF_2_- and –CF_3_ groups) undergo no obvious changes either under UV illumination or high temperature exposure. The change in transmittance for selected transitions are listed in Figure 3b, which shows that the change of –OH in TiO_2_ is much larger than the change of –CF_2_- or –CF_3_ in PFOS. The change of –CF_2_- or –CF_3_ can be observed after long time UV illumination as shown in Figure 4. Finally, PFOS was completely removed due to UV illumination and the chemical bonding between TiO_2_ and PFOS (Si–O–Ti) is without exception. The Al_2_O_3_–PFOS samples achieved the transition to super-wettability under UV illumination but required a much longer time of up to 90 min. The bands at 1210 cm^−1^ and 1240 cm^−1^ which represent the vibration of the –CF_2_- and –CF_3_ groups in PFOS was not recovered, while the –OH vibration in Al_2_O_3_ experienced no significant change.

**Figure 5:**
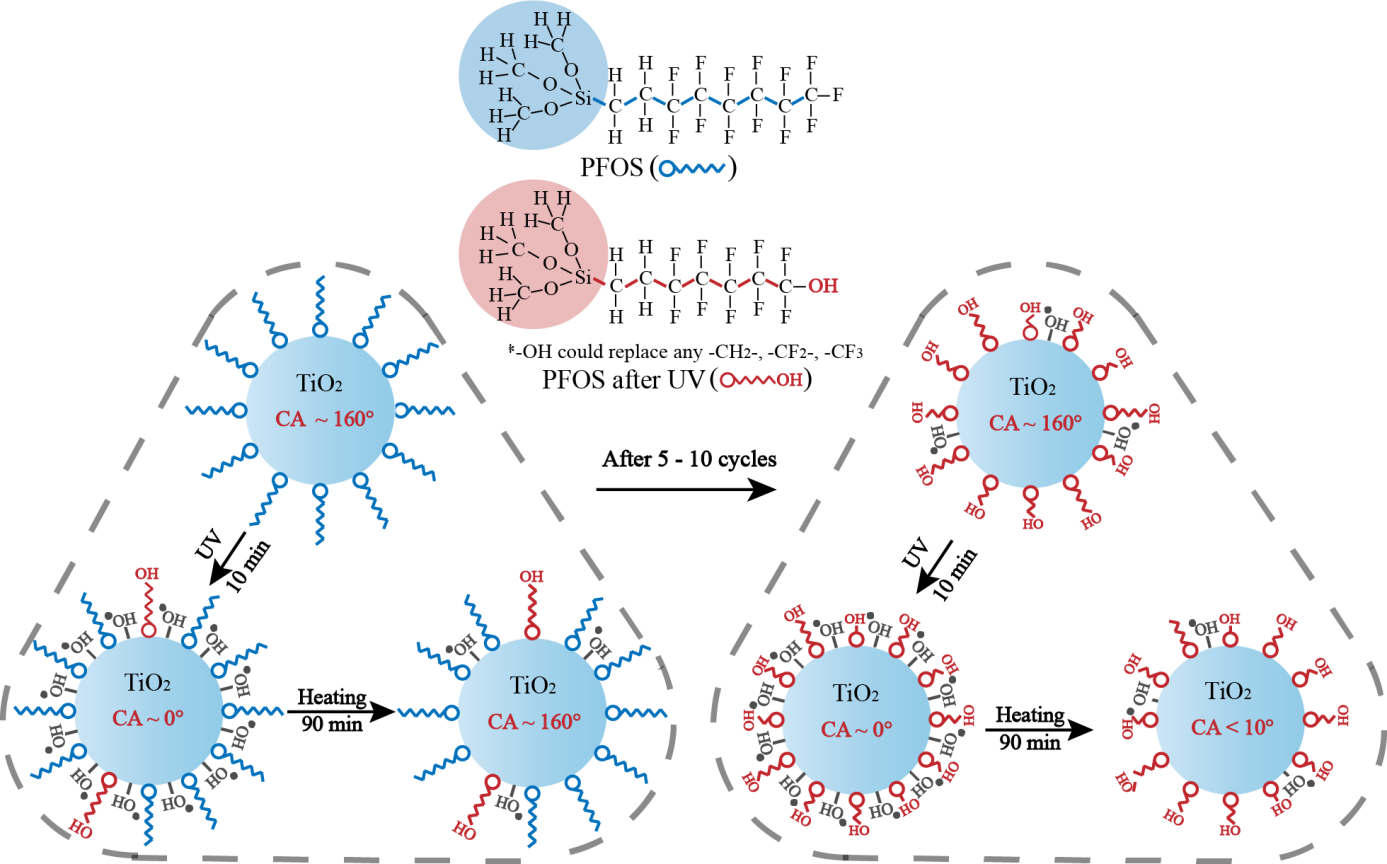
The possible mechanism describing the transition of TiO_2_ and PFOS under UV illumination and heating.

The wettability transition of TiO_2_ under UV illumination and heat treatment has been previously reported and was explained by the formation of Ti–O–H bonds under UV light, while heating of the surface results in the decrease in the concentration of Ti–O–H bonds [33–34]. As reported, due to the low band gap energy of TiO_2_, the photo-induced electron–hole pairs are generated on the TiO_2_ surfaces under UV illumination. The holes lead to the production of oxygen vacancies to enhance the adsorption of hydroxy groups, while the hydroxy groups are replaced by oxygen atoms that have a stronger bond on the defect sites during heating process. However, the change in TiO_2_ cannot fully explain the fast wettability transition of the TiO_2_–PFOS coated surface. Based on the data shown in Figure 3 and Figure 4, the –CF_2_- and –CF_3_ groups of PFOS were degraded and oxidized due to the high energy of UV illumination. The oxygen in the air stimulates a metastable state which has a strong oxidizing property. In addition, the end of the fluorocarbon chains were changed into –OH groups which could decrease the WCA faster with increasing UV illumination time. It can thus be inferred that the oxidization of PFOS is due to UV illumination and the photocatalytic effect of TiO_2_. Unlike the changes occurring in TiO_2_, unfortunately, this change in PFOS is not reversible. The mechanism for the wettability transition of the TiO_2_–PFOS coated surface under UV illumination and heating treatment is shown in Figure 5. Since the change in PFOS is not reversible, the wettability transition cycles cannot continue indefinitely, and the surface will remain superhydrophilic when most of the –CF_2_- and –CF_3_ groups are replaced by the –OH groups on PFOS. PFOS has a short carbon chain which can enhance the change of the –CF_2_- and –CF_3_ groups and results in the much faster conversion time from superhydrophobicity to superhydrophilicity compared with the previous works [35–36]. The irreversible replacement of –CF_2_- and –CF_3_ groups by –OH groups on PFOS was also confirmed by the wettability transition of the Al_2_O_3_–PFOS coated surfaces.

### Limitation of switchable cycles

To further study the wettability switching of the surfaces, four samples comprised of a uniform weight of TiO_2_ modified by different concentrations of PFOS were sprayed onto the glass samples. All the coated surfaces were initially superhydrophobic. Then the coated samples were placed under UV illumination until they transitioned to superhydrophilic, and were thereafter heated in an air-dry oven until they recovered their superhydrophobic property – this cycle was repeated until the surfaces could no longer recover the superhydrophobic property. For each concentration of PFOS, five identical samples were prepared for statistical reliability. The experimental results are shown in Table 1, where it can be seen that the number of cycles first increases with the concentration of PFOS, then decreases, while the switching time from superhydrophobic to superhydrophilic increases constantly with increasing PFOS concentration. This phenomena indicates that more PFOS can provide more –CF_2_-, –CF_3_ and can initially increase the maximum number of conversion cycles. However, an excess of PFOS results in an increase in the time to achieve superhydrophilicity which enhances the amount of –CF_2_-, –CF_3_ changed in one cycle, and subsequently decreases the number of possible cycles. As a result, to obtain a faster wettability transition time as well as a larger number of conversion cycles, an appropriate amount of PFOS and TiO_2_ is needed. It should be noted that with the same concentration of PFOS, the total UV illumination time for the surface to transition to unrecovered superhydrophilicity for TiO_2_–PFOS and Al_2_O_3_–PFOS is 30 min and 90 min, respectively. This indicates that the oxidization time of PFOS is increased by the photocatalytic effect of TiO_2_.

**Table 1:** Effect of PFOS concentration on the wettability conversion time and number of conversion cycles.

Amount of PFOS in 50 mL ethanol (g)	Initial contact angle (°)	*R*_a_ (μm)	Wettability conversion time (min)	Number of cycles

0.5	169	2.334	5	0–1
1.0	171	2.627	10	2–3
1.5	172	2.550	30	5–6
2.0	168	2.877	60	3–4

**Figure 6:**
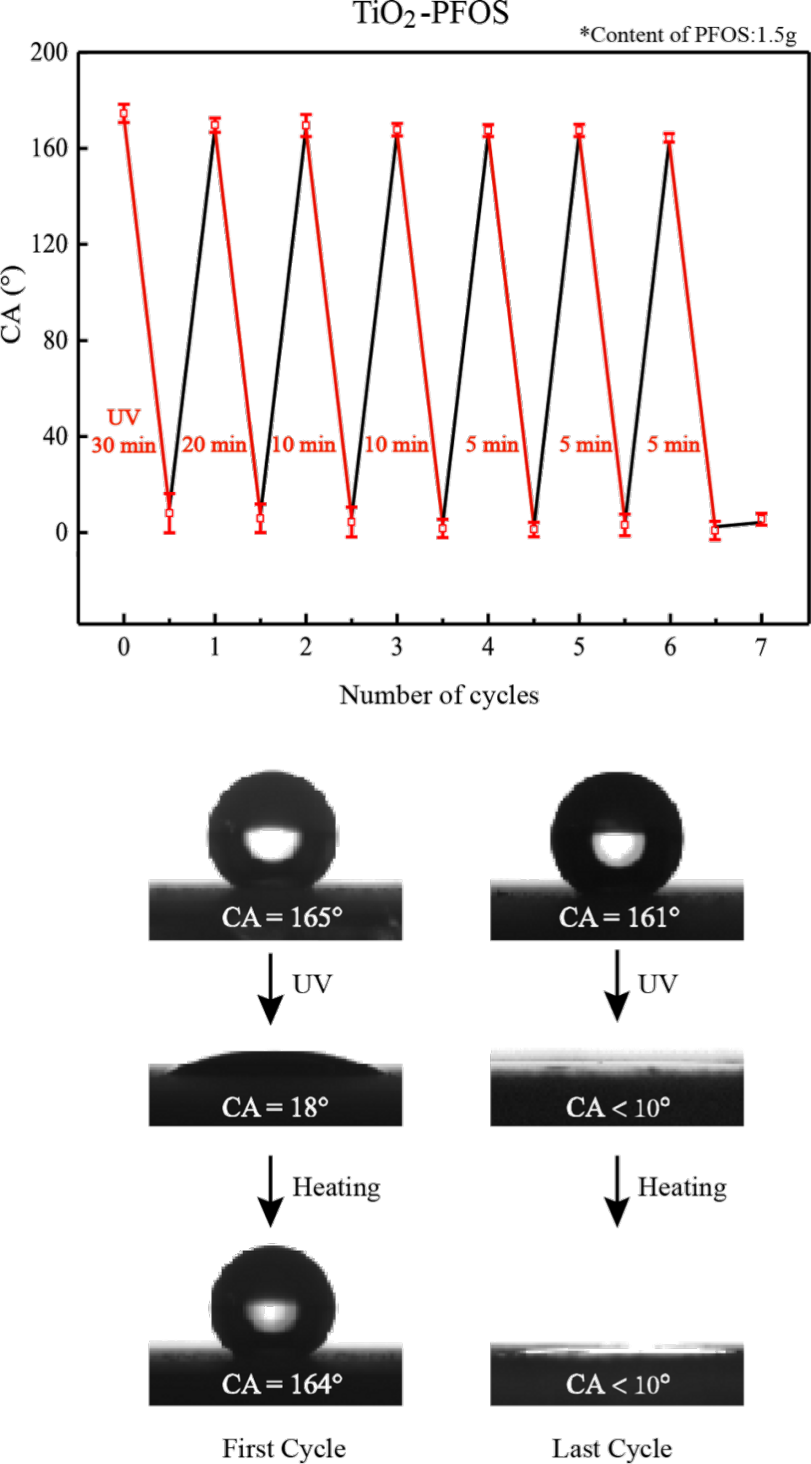
Reversible superhydrophobic/superhydrophilic switching of the composite surface under UV illumination and heating (top), and the first and last cycle wettability switching process of a sample (bottom).

For the sample with 1.5 g PFOS, the CA of the six cycles and the images of the conversion processes in the first and the last cycles are shown in Figure 6. The transition time from superhydrophobic to superhydrophilic decreased from the first cycle to the last cycle. This can be explained by the unrecoverable dissociation of the –CF_2_- and –CF_3_ groups, which is also in agreement with the proposed mechanism in Figure 5.

## Conclusion

TiO_2_ nanoparticles modified with PFOS (which has relatively short carbon chains) were applied on glass substrates to achieve fast, switchable super-wettability, demonstrated by a WCA that could be recovered from ≈165° to almost 0°. The experimental results show that the PFOS could reduce the conversion time from nearly 1 h to about 10 min, and the superhydrophobicity of the surfaces could be recovered after heating for 90 min at 120 °C. However, it was shown that certain concentrations of PFOS limit the number of recovery cycles, whereby the optimal concentration of PFOS was identified to achieve about 5 to 10 cycles. The unrecoverable change of the –CF_2_- and –CF_3_ groups on PFOS and the recoverable change of –OH groups on TiO_2_ were recorded by FTIR. These effects can explain both the shorter transition time and the limitation that (initially) the change of the –CF_2_- and –CF_3_ groups leads to a quicker conversion time from superhydrophobicity to superhydrophilicity. However, this is only true when most of the –CF_2_- and –CF_3_ groups are changed unrecoverably, and the whole surface cannot not transition back to a superhydrophobic state.

## Experimental

### Materials and chemicals

Titanium dioxide (TiO_2_, rutile) with a diameter of ≈25 nm and aluminium dioxide (Al_2_O_3_) with a diameter of ≈30 nm were acquired from Shanghai Aladdin Bio-Chem Technology Corporation (Shanghai, China). 1*H*,1*H*,2*H*,2*H*-Perfluorooctyl(trimethoxy)silane (PFOS) was obtained from Shanghai Macklin Biochemical Corporation (Shanghai, China). Other chemicals include 99.9% ethanol used as the solvent and deionized water was used in the CA measurements.

### Preparation of coating surfaces

Rutile phase TiO_2_ was modified by a simple hydrolysis reaction in order to obtain superhydrophobicity. In the modification process, 1 g of PFOS was dissolved into 50 mL ethanol and stirred for 1 h at ambient temperature in order to fully hydrolyse fluoroalkylsilane. Next, 3 g of rutile TiO_2_ was added into the solution and stirred for another 1 h to form the suspension. The suspension could be used to spray or paint a substrate with a spray gun (20 kPa). When the surfaces were dried, the superhydrophobicity is revealed. Al_2_O_3_ was modified by the same method as TiO_2_.

### UV illumination and heating experiments

A UV lamp (30 W) obtained from Cnlight Optical-Electrical Technology Corporation (Guangdong, China) was used to generate the UV illumination. The ultraviolet power density was set at 94 µW/cm^2^ and the wavelength was 254 nm. The change of the WCA under UV light illumination was measured at short intervals. For the heating process, which was employed to recover the superhydrophobicity, the surface was heated in the air-dry oven at 150 °C for 90 min after UV illumination. The switching time from superhydrophilic to superhydrophobic was found to be reduced with increasing heating temperature.

### Characterization

The microstructure of the coating surfaces was studied using scanning electron microscopy (SEM, ZEISS MERLIN Compact SEM, operated at a 20 kV acceleration voltage, Carl Zeiss Jena, Germany), and the surface roughness was measured with a laser confocal microscope (LCM, OLS5000, Olympus, Japan). The chemical modification and the end group changes on the surfaces was studied by Fourier transform infrared spectroscopy (FTIR, Nicolet iS50, Thermo Scientific, USA). The contact angles were measured at room temperature with an optical contact angle meter (DropMeterTM Element A-60, Maist, Ningbo, China), where the static CAs of the droplets (6 µL) placed onto the surfaces were measured five times at different locations.
